# Comparison of Three Cellular Assays to Predict the Course of CMV Infection in Liver Transplant Recipients

**DOI:** 10.3390/vaccines9020088

**Published:** 2021-01-25

**Authors:** Smaranda Gliga, Melanie Fiedler, Theresa Dornieden, Anne Achterfeld, Andreas Paul, Peter A. Horn, Kerstin Herzer, Monika Lindemann

**Affiliations:** 1 Institute for Transfusion Medicine, University Hospital Essen, Virchowstraße 179, 45147 Essen, Germany; smaranda.gliga@gmail.com (S.G.); theresa.dornieden@outlook.de (T.D.); peter.horn@uk-essen.de (P.A.H.); 2 Institute for Virology, University Hospital Essen, University Essen-Duisburg, 45147 Essen, Germany; melanie.fiedler@uk-essen.de; 3 Department of Gastroenterology and Hepatology, University Hospital Essen, University Essen-Duisburg, 45147 Essen, Germany; anne.achterfeld@uk-essen.de (A.A.); kerstin.herzer@uk-essen.de (K.H.); 4 Department of General, Visceral and Transplantation Surgery, University Hospital Essen, University Essen-Duisburg, 45147 Essen, Germany; andreas.paul@uk-essen.de; 5 Knappschaftsklinik Bad Neuenahr, 53474 Bad Neuenahr-Ahrweiler, Germany

**Keywords:** human *cytomegalovirus*, reactivation, ELISpot, ELISA, interferon-γ, liver transplantation, prediction

## Abstract

To estimate protection from *cytomegalovirus* (CMV) replication after solid organ transplantation, CMV serology has been considered insufficient and thus CMV immunity is increasingly assessed by cellular in vitro methods. We compared two commercially available IFN-γ ELISpot assays (T-Track CMV and T-SPOT*.CMV*) and an IFN-γ ELISA (QuantiFERON-CMV). Currently, there is no study comparing these three assays. The assays were performed in 56 liver transplant recipients at the end of antiviral prophylaxis and one month thereafter. In CMV high- or intermediate-risk patients the two ELISpot assays showed significant correlation (*p* < 0.0001, *r* > 0.6) but the correlation of the ELISpot assays with QuantiFERON-CMV was weaker. Results of both ELISpot assays were similarly predictive of protection from CMV-DNAemia ≥500 copies/mL [CMV pp65 T-SPOT.*CMV* at the end of prophylaxis: area under curve (AUC) = 0.744, cut-off 142 spot forming units (SFU), sensitivity set to 100%, specificity 46%; CMV IE-1 T-Track CMV at month 1: AUC = 0.762, cut-off 3.5 SFU, sensitivity set to 100%, specificity 59%]. The QuantiFERON-CMV assay was inferior, reaching a specificity of 23% when setting the sensitivity to 100%. In conclusion, both CMV-specific ELISpot assays appear suitable to assess protection from CMV infection/reactivation in liver transplant recipients.

## 1. Introduction

The seroprevalence of the human cytomegalovirus (CMV), a DNA virus of the herpes family, ranges between 45 and 100% in the general adult population [1]. In immunocompetent individuals, the course of the infection is usually asymptomatic or mild, with symptoms of mononucleosis [2]. In contrast, in transplant recipients CMV infection or reactivation can lead to fever, hepatitis, colitis, interstitial pneumonitis, encephalitis, vasculopathy or allograft rejection [3,4,5]. In these immunocompromised individuals CMV infection/reactivation results in significant morbidity and treatment costs. It can even be life-threatening [3]. Furthermore, in liver transplant recipients it was associated with a higher rate of accelerated hepatitis C recurrence, hepatic artery thrombosis and cholangitis [6,7].

Without antiviral prophylaxis, up to 75% of solid organ transplant recipients suffer from CMV infection/reactivation [8] and 18–29% develop CMV disease, usually during the first three months after transplantation [9]. Whereas infection/reactivation is defined by viremia, CMV disease is defined by viremia and clinical symptoms. There are currently two strategies to prevent CMV infection/reactivation, prophylactic administration of antiviral drugs or pre-emptive treatment, i.e., regular monitoring for CMV-DNA and administration of antiviral therapy as soon as viral load reaches a certain threshold [8,9]. With these strategies the incidence of CMV disease can be nearly halved and the onset of disease occurs later, usually during the first three–six months after completing antiviral prophylaxis [9]. Patients at high risk of developing CMV infection and disease [donor (D)+/recipient (R)- CMV-IgG serostatus prior to transplantation] and intermediate-risk patients (D-/R+, D+/R+) usually receive antiviral prophylaxis at our transplant center. However, other centers use pre-emptive treatment in intermediate-risk patients, which is considered similarly effective in preventing CMV disease in this patient group [9].

In solid organ transplant recipients CMV serology has been considered insufficient to predict CMV-DNAemia [10]. However, within recent years it has been shown that the quantification of cellular in vitro responses against CMV can help to stratify the risk of CMV infection/reactivation [11,12,13,14,15,16,17,18,19,20,21,22,23,24]. The application of a highly sensitive method, the CMV-specific ELISpot, which can detect cytokine secretion on a single cell level [25], has been described in several cohorts of kidney transplant recipients. It may help to guide prophylactic or pre-emptive antiviral treatment [11,12,13,16,22]. In contrast, data on liver transplant recipients are scarce and we are aware of only one recent report on a CMV-specific ELISpot in this cohort [17]. This study included 32 liver transplant recipients and used a CMV-specific interferon (IFN)-γ ELISpot assay (CMVspot, Autoimmun Diagnostika, Straßberg, Germany), performed one day before transplant surgery.

In the current study, we focused on one transplanted organ (liver) and one clinical scenario (CMV-DNAemia after discontinuation of prophylaxis). The primary endpoint was comparing three CMV-specific Interferon-Gamma-Release Assays (IGRAs), two CE-marked, commercially available CMV ELISpot assays (T-Track CMV, Lophius Biosciences (Lo) (Regensburg, Germany) and T-SPOT*.CMV*, Oxford Immunotec (OI) (Abingdon, Oxfordshire, UK) and a CMV-IFN-γ ELISA (QuantiFERON-CMV, QIAGEN, Hilden, Germany)). Of note, both ELISpot assays detect CMV-specific responses of CD4+ and CD8+ T cells and the T-Track CMV furthermore measures the response of activated NK and NKT-like cells [16,26]. In contrast, the QuantiFERON-CMV assay only detects CD8+ T cells [16]; which could affect the predictive value of the various assays. Secondarily, we compared cellular responses at the end of antiviral prophylaxis with results one month thereafter. Thirdly, we determined cut-off values for cellular responses that protect against CMV infection/reactivation. Finally, the strength of CMV-specific cellular immunity was correlated with patient demographics.

## 2. Materials and Methods

### 2.1. Patients

Fifty-six adult liver transplant recipients were enrolled consecutively in this study from April 2016 to July 2018 (Table 1). The patients were very predominantly of Caucasian origin. The high-risk patients received antiviral prophylaxis for 200 days, the intermediate-risk patients for 100 days (450 mg valganciclovir twice a day). Patients with the D−R− constellation were excluded. The two ELISpot assays were performed immediately after the end of prophylaxis (same day) and one month thereafter. The QuantiFERON-CMV assay was performed parallel to the ELISpot and was continued usually monthly until month 6 after the end of prophylaxis. In addition, CMV-DNA was quantified monthly by PCR. For the evaluation of the CMV-DNAemia, we used two different cut-offs [≥40 copies/mL (≥62.4 IU/mL) or ≥500 copies/mL (≥780 IU/mL)]. The first cut-off is the detection limit of the assay and the second one is the viral load considered as substantial. At the end of prophylaxis, the majority of patients received immunosuppressive therapy with a calcineurin inhibitor (tacrolimus) and mycophenolate mofetil (MMF), as detailed in Table 1. During the follow-up of six months, the treatment in 34 patients was switched from MMF to an mTOR inhibitor (everolimus). Usually, the switch was performed at month 3, coupled with a discontinuation in steroid administration. Furthermore, in one patient without previous MMF, everolimus was newly administered. Eighteen patients with CMV infection/reactivation received antiviral therapy. Oral valganciclovir (900 mg twice a day, adapted to the kidney function) was preferred. Four patients displaying detectable CMV-DNAemia only once were left without antiviral treatment. The study was approved by our institutional review board (ethics committee of the University Hospital Essen, approval no. 15-6738-BO) and was carried out in accordance with the code of ethics of the World Medical Association (Declaration of Helsinki and Declaration of Istanbul). Written informed consent was obtained from each participant.

### 2.2. ELISpot and QuantiFERON Assays

Concurrently, 9 mL blood was collected in heparin tubes for the ELISpot assays and 3 mL blood was collected in three QuantiFERON tubes (1 mL each, negative control, CMV peptides and positive control). For the T-Track CMV, PBMC were isolated and adjusted to 2 million lymphocytes per milliliter [12]. Two-hundred thousand lymphocytes were added to each well of 8-well ELISpot strips and stimulated for 19 h at 37 °C in quadruplicates with two T-activated^®^ CMV proteins, immediate early antigen-1 (IE-1) and phosphoprotein 65 (pp65), according to the manufacturer’s instructions. In parallel, negative controls (cells with medium only) and positive controls (stimulated with the mitogen phytohemagglutinin) were cultured. The results were generated according to an algorithm provided by the manufacturer for the evaluation of four replicate values or considering median values (which is easier to calculate). Using the provided algorithm, the arithmetic mean of square-root-transformed 4-replicate spot counts is calculated and squared. If not further specified, results were generated using the manufacturer’s algorithm. For the T-SPOT.CMV, 250,000 PBMC were seeded in each of four wells of 8-well strips, according to the manufacturer’s instructions. The cells were stimulated for 19 h at 37 °C with CMV-specific peptides (IE-1 and pp65), parallel with negative and positive controls.

For both ELISpot assays, the resultant spots, each representing a single IFN-γ releasing cell, were quantified using an ELISpot plate reader (AID Fluorospot, Autoimmun Diagnostika GmbH, Strassberg, Germany). Negative controls were subtracted from CMV-specific values, resulting in spot forming units (SFU). SFU to the T-Track CMV (normalized to 200,000 lymphocytes) or T-SPOT.CMV (normalized to 250,000 PBMC) were defined as positive at ≥10 SFU, respectively. Of note, the manufacturer of the T-SPOT.CMV (OI) does not provide a cut-off for positivity. However, to compare the data we have chosen the same cut-off for both ELISpot assays.

The QuantiFERON tubes were incubated for 19 h at 37 °C and processed according to the manufacturer’s instructions. The CMV peptides are mapped within pp65, IE-1, pp50, IE-2, gB and pp28 and their presentation is restricted to prevailing human leukocyte antigen (HLA) class I molecules (present in 98% of the Caucasian population) [27]. The QuantiFERON-CMV results were considered positive at ≥0.2 IU/mL IFN-γ.

### 2.3. CMV Status and Viral Load

CMV-IgG was determined prior to transplantation using the Anti-CMV-IgG^®^ (DiaSorin, Saluggia, Italy) assay on the LIASON XL platform, following the manufacturer’s instructions. In addition, an Anti-CMV-IgM^®^ (DiaSorin, Saluggia, Italy ) was used to detect CMV primary infection. Using this platform, CMV-IgG <12 IU/mL is considered negative, from 12 to 14 IU/mL borderline and >14 IU/mL positive. CMV-DNA was purified from whole blood samples using the Abbott m2000sp automated nucleic acid extraction system (Abbott, Wiesbaden, Germany) and quantified with the full-automated Abbott m2000rt real-time PCR system using the Abbott RealTime CMV amplification reagent kit, according to the manufacturer’s instructions. The detection limit is 40 copies/mL (62.4 IU/mL).

### 2.4. Statistics

Statistical analysis was performed using GraphPad Prism 8.0.1 (San Diego, CA, USA) and IBM SPSS Statistics 23 (New York, NY, USA) software. Correlation of cellular responses in various assays was determined by Spearman correlation and linear regression analysis. Results at the end of the prophylaxis and at month 1 or results of ELISpot assays from two companies were compared by Wilcoxon matched pairs test. For the comparison of two patient groups the Mann-Whitney U test was used. The predictive value of the IGRAs was evaluated by receiver operating characteristic (ROC) curve analysis. The endpoint was protection against the emergence of any CMV-DNAemia (i.e., ≥40 copies/mL) or of substantial CMV-DNAemia (≥500 copies/mL) within six months after the end of prophylaxis. To determine sensitivity and specificity, cellular reactions above a certain cut-off were defined as predictors of freedom from CMV-DNAemia (infection/reactivation). Using this definition, a sensitivity of 100% means that all patients with infection/reactivation show responses below this cut-off. Thus, none of the patients with CMV-DNAemia would be missed. Consequently, responses above the cut-off only occur in patients with freedom from CMV-DNAemia. A specificity of e.g., 50% means that half of the patients without CMV-DNAemia are classified correctly as protected and reach responses above the cut-off. To assess the correlation of CMV-specific cellular responses with additional clinical parameters, Spearman correlation analysis, Mann-Whitney test or Kruskal-Wallis test was used as appropriate. Two-sided *p* values <0.05 were considered significant.

## 3. Results

### 3.1. Correlation of Three CMV-Specific Interferon-Gamma-Release Assays

In 56 liver transplant recipients belonging to the high- or intermediate-risk group for CMV, three CMV-specific IGRAs were compared. Details on the study cohort are summarized in Table 1. Of note, 22 out of 56 patients (39%) developed CMV-DNAemia until six months after the end of prophylaxis.

**Figure 1:**
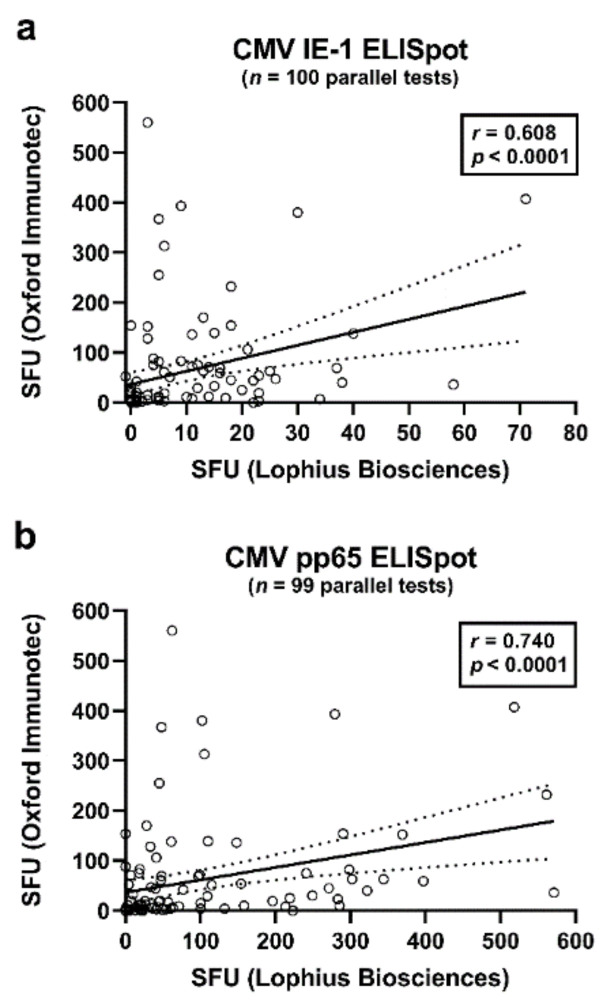
Correlation of CMV-specific ELISpot responses of the T-Track CMV (Lophius Biosciences) and of the T-SPOT.*CMV* (Oxford Immunotec). This analysis included all pairs of data sets (parallel tests with T-Track CMV and T-SPOT.*CMV*) in liver transplant recipients (50 at the end of prophylaxis and 49–50 at month 1 thereafter), as also shown in Table 2. Panel (**a**) shows interferon-γ responses to CMV IE-1 antigens (*n* = 100) and panel (**b**) to CMV pp65 antigens (*n* = 99). At both time points, six out of 56 patients were not tested in parallel. The bold, continuous line indicates the regression line, the dashed lines the 95% confidence interval. SFU—spot forming units.

The Spearman correlation analysis included results of the T-SPOT*.CMV* (OI), T-Track CMV (Lo) and QuantiFERON-CMV assays. It considered data at the end of antiviral prophylaxis and one month thereafter. Correlation of results of two ELISpot assays at a given time point was highly significant (*p <* 0.0001). CMV IE-1-specific responses of the T-Track CMV—as determined by the algorithm of the manufacturer—and T-SPOT*.CMV* correlated moderately (*r =* 0.608), while CMV pp65-specific responses correlated strongly (*r =* 0.740) (Figure 1, Table 2a, marked bold). The algorithm as specified by the manufacturer Lophius Biosciences (detailed in the Materials and Methods section) or the use of the median value of quadruplicate cell cultures yielded very similar results (*r* = 0.987 to *r* = 0.996) (Table 2a, marked in italics). Furthermore, ELISpot responses to the two antigens, IE-1 and pp65, showed moderate to strong correlations (*r* = 0.609 to *r* = 0.706) (Table 2a).

Results of both ELISpot assays showed weak to moderate correlations with results of the QuantiFERON-CMV assay (Table 2b); correlation with CMV IE-1-specific ELISpot assays was weak (*r =* 0.217 to *r =* 0.387) and correlation with CMV pp65-specific ELISpot assays was moderate (*r =* 0.551 to *r =* 0.647).

### 3.2. Comparison of Cellular Responses at the End of Antiviral Prophylaxis with Results One Month Thereafter

**Figure 2:**
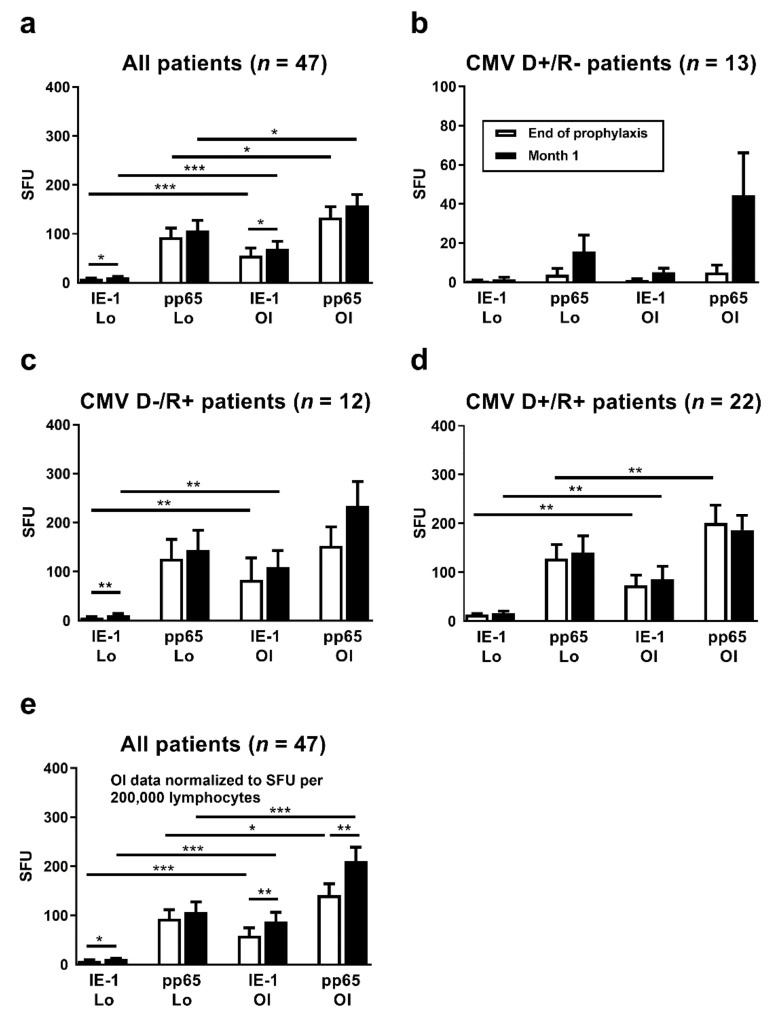
Comparison of CMV-specific ELISpot responses at the end of antiviral prophylaxis (white bars) and at month 1 thereafter (black bars). Results of the T-Track CMV (Lophius Biosciences, Lo) and of the T-SPOT.*CMV* (Oxford Immunotec, OI) in liver transplant recipients are shown as spot forming units (SFU) per 200,000 lymphocytes (T-Track CMV) and per 250,000 PBMC (T-SPOT.*CMV*) in panel (**a**–**d**). To allow a better comparison of the strength of reactions, results of the T-SPOT.*CMV* were further normalized to lymphocyte numbers and given as SFU per 200,000 lymphocytes, as shown in panel (**e**). Results were only considered if datasets for both ELISpot assays at the end of prophylaxis and at month 1 were available (47 out of 56 patients). Panel (**a**) and (**e**) show data on all liver transplant recipients (*n* = 47), panel (**b**–**d**) on patients with various combinations of CMV IgG in donors (D) and recipients (R). Please note the different scale on the y axis in panel (**b**). Mean and standard of the mean (SEM) are indicated. Data were compared by Wilcoxon matched pairs test (* *p* < 0.05, ** *p* < 0.01, *** *p* < 0.001).

**Figure 3:**
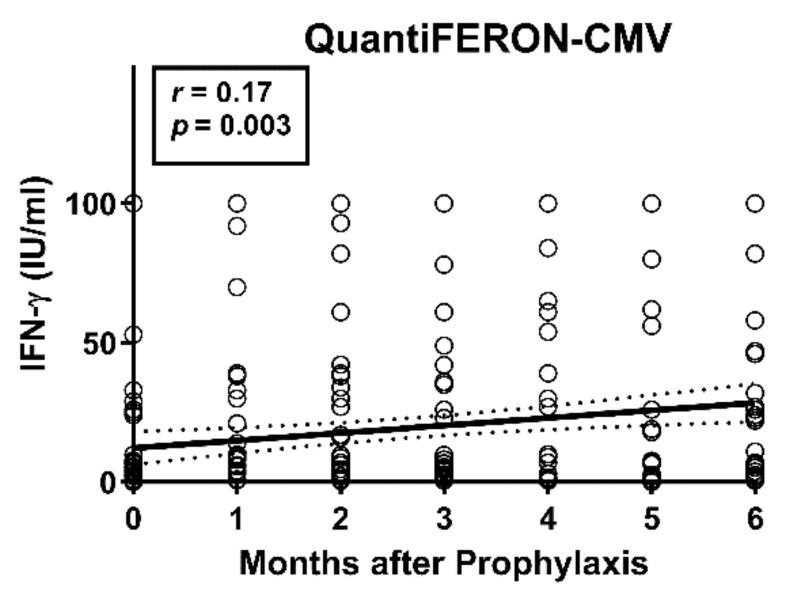
Course of responses in the QuantiFERON-CMV assay in 56 liver transplant recipients. Results of the QuantiFERON-CMV assay were analyzed from the end of antiviral prophylaxis until six months thereafter. The time after the end of prophylaxis correlated significantly with the concentration of IFN-γ, as analyzed by Spearman correlation analysis. The bold, continuous line indicates the regression line, the dashed lines the 95% confidence interval.

Between the end of antiviral prophylaxis and one month thereafter, CMV IE-1-specific ELISpot responses increased 1.4-fold (T-Track CMV, Lo) or 1.2-fold (T-SPOT*.CMV,* OI), respectively (*p <* 0.05), considering 47 liver transplant patients with data sets for both ELISpot assays at both time points (Figure 2a). Increases in CMV pp65-specific responses, however, were non-significant. For further analysis, the patients were subdivided according to their CMV-IgG serostatus prior to transplantation. The high-risk patients (D+/R−) showed the largest increase in cellular immune responses between the two time points, on a percentage basis (Figure 2b). In these high-risk patients CMV IE-1-specific ELISpot responses increased 2.0-fold (T-Track CMV, Lo) or 4.5-fold (T-SPOT*.CMV,* OI), respectively, and CMV pp65-specific ELISpot responses increased 4.1-fold (T-Track CMV, Lo) or 9.0-fold (T-SPOT*.CMV,* OI), respectively. However, changes were non-significant. D−/R+ patients showed an intermediate increase of cellular responses (Figure 2c). In the D+/R+ group only a minor increase of CMV IE-1-specific responses could be observed, whereas CMV pp65-specific responses remained unchanged (Figure 2d). Results of the T-SPOT*.CMV* (OI, expressed as SFU/250,000 PBMC) were significantly higher (*p* < 0.05) than those to the T-Track CMV (Lo, expressed as SFU/200,000 lymphocytes), both at the end of prophylaxis and at month 1 thereafter (Figure 2a,c,d). To allow a better comparison of the strength of reactions, results of the T-SPOT.*CMV* were normalized to lymphocyte numbers (Figure 2e). When considered as SFU/200,000 lymphocytes, differences between the two ELISpot assays were even more pronounced. Similar to the ELISpot results, we observed a time-dependent increase of IFN-γ responses with the QuantiFERON-CMV assay (Figure 3).

In eight out of 14 high-risk D+/R− patients CMV DNA was detectable within six months after the end of prophylaxis (Table 3, Supplementary Table S1). Of note, none of the high-risk patients tested positive between transplantation and the end of prophylaxis. In three patients, CMV DNA was detected for the first time at month one, in three patients at month two and in two patients at month three after end of prophylaxis. In three patients (ID 6–8), both ELISpot assays and the QuantiFERON-CMV assay were positive prior to the first detection of any CMV-DNAemia (marked green). These patients contributed substantially to the increase of CMV-specific cellular immunity at month one, as also shown in Figure 2b. Of note, in these three patients the maximum viral load was below 500 copies/mL. Moreover, in one patient (ID 1) both ELISpot assays and the QuantiFERON-CMV assay were positive at the same time CMV-DNAemia was detected (marked yellow). In another patient (ID 5), only the T-SPOT.CMV (OI) was positive concomitantly to the detection of CMV-DNAemia (also marked yellow), but the T-Track CMV (Lo) was not. In this patient, the QuantiFERON-CMV turned positive later than the viral load (marked orange). In a further patient with the onset of CMV-DNAemia at month three (ID 3), the QuantiFERON-CMV turned positive at month two (marked green). Altogether, in four out of eight patients with CMV replication (ID 3, 6–8) the positivity to the IGRAs preceded CMV-DNAemia.

### 3.3. Determination of Cut-off Values for Protective T Cell Responses

**Figure 4:**
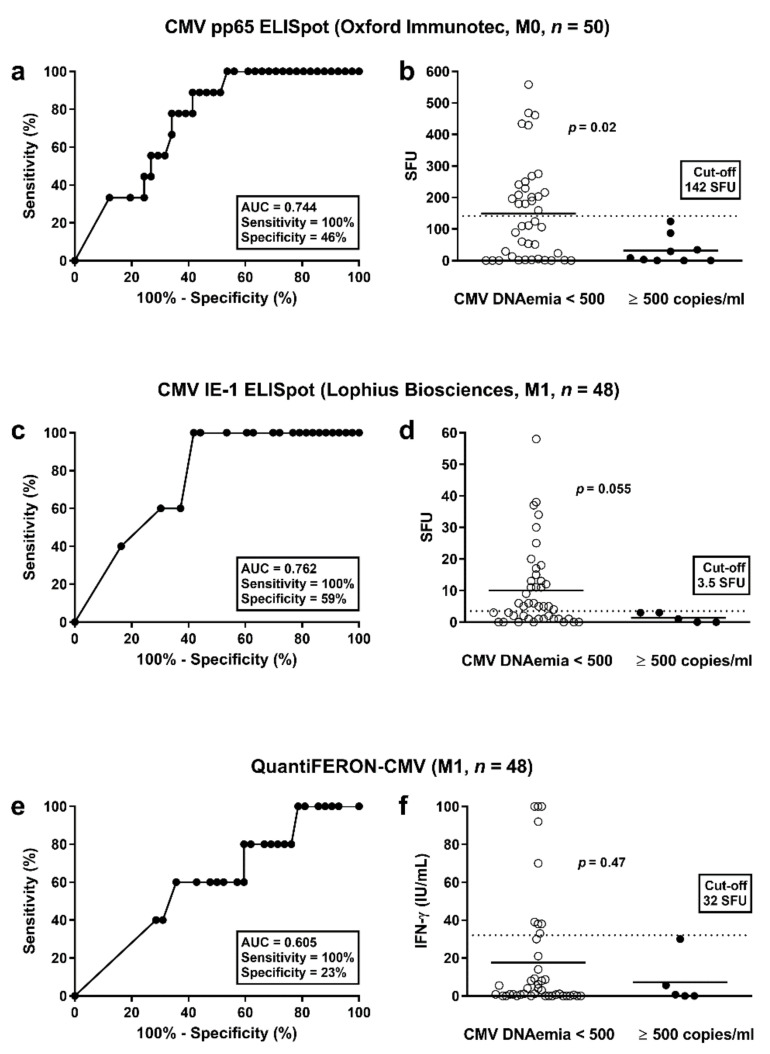
Discrimination between patients without and with CMV-DNAemia. This analysis considered CMV pp65-specific responses of the T-SPOT.*CMV* at the end of prophylaxis (**a**,**b**) and CMV IE-1-specific responses of the T-Track CMV at month 1 after the end of antiviral prophylaxis (**c**,**d**). For comparison, results of the QuantiFERON-CMV assay were shown (**e**,**f**). The cut-off for CMV-DNAemia was set at 500 copies/mL (substantial DNAemia). Panel (**a**,**c**,**e**) show results of receiver operating characteristic (ROC) curve analyses. It was analyzed if ELISpot or ELISA results were predictive of significant CMV-DNAemia. Panel (**b**,**d**,**f**) compare responses of the CMV pp65-ELISpot, CMV IE-1-ELISpot and QuantiFERON-CMV assay in patients with and without substantial CMV-DNAemia (Mann-Whitney *U* test). The horizontal lines indicate the mean values, the dashed line cut-off values as defined by ROC curve analyses (142 spot forming units (SFU), 3.5 SFU and 32 IU/mL, respectively). M = months after the end of prophylaxis.

To determine whether the strength of cellular responses towards CMV was predictive of subsequent protection from CMV infection/reactivation, ROC curve analyses were performed. Protection was either defined as absence from any detectable CMV-DNAemia (<40 copies/mL) or as absence from substantial CMV-DNAemia (<500 copies/mL), i.e., we used two cut-offs for calculation. We set the sensitivity to 100% in order not to miss any of the patients with CMV-DNAemia. Consequently, all patients with responses above a certain cut-off will be certainly protected from CMV infection/reactivation. We performed two analyses, one at the end of prophylaxis and a second one month thereafter. Patients who developed CMV infection/reactivation between the end of prophylaxis and month one were excluded from the ROC curve analysis at month one. All cellular assays performed better in predicting protection from substantial CMV-DNAemia (≥500 copies/mL) than in predicting protection from any detectable CMV-DNAemia (≥40 copies/mL) (Supplementary Table S2). At the end of prophylaxis (month 0), CMV pp65-specific responses of the T-SPOT.*CMV* (OI) were the best predictor of protection from substantial CMV-DNAemia [area under curve (AUC) = 0.744; cut-off 142 SFU, sensitivity set to 100%, specificity 46%] (Figure 4a, Table 4, Supplementary Tables S2 and S3). In all patients with substantial CMV-DNAemia results the ELISpot were below the cut-off of 142 SFU (Figure 4b). Vice versa, all patients with results exceeding this cut-off did not display substantial CMV-DNAemia. Patients with vs. without CMV infection/reactivation had a mean value of 32 vs. 149 SFU/250,000 PBMC (*p =* 0.02). At month one, CMV IE-1-specific responses of the T-Track CMV (Lo) were the best predictor of protection from substantial CMV-DNAemia (AUC = 0.762; cut-off 3.5 SFU, sensitivity set to 100%, specificity 59% (Figure 4c, Table 4, Supplementary Tables S2 and S3). Patients with vs. without CMV infection/reactivation had a mean value of 1.4 vs. 10.1 SFU/200,000 lymphocytes (*p =* 0.055) (Figure 4d). Responses of the QuantiFERON-CMV assay were less predictive (e.g., at month 1: AUC = 0.605; cut-off 32 IU/mL, sensitivity set to 100%, specificity 23% (Figure 4e, Table 4, Supplementary Tables S2 and S3). Patients with vs. without CMV infection/reactivation had a mean value of 4.3 vs. 17.6 IU/mL (*p =* 0.47) (Figure 4f).

We analyzed the D+/R− subgroup separately (lower panel of Table 4 and Supplementary Tables S2 and S3). At the end of prophylaxis, CMV pp65-specific responses of the T-Track CMV (Lo) were the best predictor of protection from substantial CMV-DNAemia (AUC = 0.700; cut-off 0.5 SFU, sensitivity set to 100%, specificity 40%). One month thereafter, CMV pp65-specific responses of the T-Track CMV (Lo) and T-SPOT*.CMV* (OI) equally predicted protection (AUC = 0.722; T-Track CMV pp65: cut-off 0.5 SFU, sensitivity set to 100%, specificity 44%; T-SPOT*.CMV* pp65: cut-off 3.5 SFU, sensitivity set to 100%, specificity 44%). Thus, both ELISpot assays were overall similarly predictive of protection from CMV infection/reactivation. As compared to the ELISpot assays, the specificity of the QuantiFERON-CMV assay was lower (33%).

### 3.4. Correlation of CMV-Specific Cellular Immunity with Additonal Clinical Parameters

We further investigated whether patient age, sex or underlying disease leading to transplantation correlated with CMV-specific cellular immunity. A significant but weak positive correlation between patient age and T-SPOT.*CMV* or T-Track CMV results was found, both at the end of prophylaxis and at one month thereafter. The highest correlation coefficient was obtained for IE-1-specific T-Track CMV at month one (*r =* 0.42 and *p =* 0.001). On the other hand, age did not correlate with QuantiFERON-CMV results (*r* < 0.12 and *p* > 0.4). The Mann-Whitney test indicated that sex did not correlate significantly with CMV-specific cellular immunity. However, females tended to react stronger to all cellular assays (*p* ≥ 0.22). The Kruskal-Wallis test showed that the disease leading to transplantation (as outlined in Table 1) did not correlate significantly (*p* ≥ 0.09) with CMV-specific cellular immunity.

## 4. Discussion

Following solid-organ transplantation, cellular assays predicting CMV replication might help to optimize patient management and treatment. In the current study, we performed three CMV-specific IGRAs in liver transplant recipients. We have chosen the end of prophylaxis and one month thereafter for the comparative measurements because the end of prophylaxis is a time point when further treatment has to be fixed and when an individualized risk-adapted treatment could be beneficial for the patient. In patients at high risk but without CMV-specific cellular immunity it may be reasonable to extend antiviral prophylaxis, whereas in patients with cellular immunity above a certain threshold close monitoring of the viral load may be sufficient. If corroborated by an interventional study, cellular immunity above this threshold may justify that antiviral prophylaxis could be omitted. Of note, at the end of prophylaxis 19 out of 50 liver transplant recipients (38%) exceed the cut-off value defined in the current cohort (142 SFU to the pp65-specific T-SPOT.*CMV,* OI) (Figure 4b, Supplementary Table S3) and none of them developed substantial CMV-DNAemia (≥500 copies/mL) within the follow-up period of six months. Moreover, at one month after the end of prophylaxis 25 out 48 of patients (52%) exceeded the cut-off (3.5 SFU to the IE-1-specific T-Track CMV, Lo) (Figure 4d, Supplementary Table S3) and none of them developed substantial CMV-DNAemia.

According to our Spearman analyses, the correlation between the two ELISpot assays was moderate (IE-1) to strong (pp65), while that between each ELISpot assay and the QuantiFERON-CMV assay was weak (IE-1) to moderate (pp65). Hence, the correlation between the two ELISpot assays was higher than between ELISpot and ELISA (QuantiFERON-CMV assay). A reduced correlation between ELISpot and ELISA results might be attributed to differences in the assay procedure, and to the restricted ability of the QuantiFERON-CMV assay to detect responses of CD8+ T cells only, as opposed to the ELISpot assays, which detect responses of both CD4+ and CD8+ T cells [16]. Flow cytometry and intracellular cytokine yielded higher percentages of CMV-specific CD8+ than CD4+ T cells [18]. Considering individuals with CMV-DNAemia prompting initiation of treatment, CMV-specific CD8+ vs. CD4+ T cell numbers were 3.5-fold higher. For comparison, in those without CMV events CD8+ vs. CD4+ T cell numbers were 1.2-fold higher [18]. Interestingly, experimental mouse models indicate that CMV infection with a high infectious dose causes immune perturbations that impair CD8+ T cell immunity later in life [28]. Specifically, a high infectious dose appeared as a prerequisite for CMV-associated compromised immunity against heterologous infection (with other viruses) and for immune senescence [28]. In humans, dysfunctional CD4+ and CD8+ T cells, as defined by loss of cytokine secretion ability and limited proliferation capacity, were observed after CMV infection [29,30,31,32]. It was described that the magnitude of the CMV-specific CD4+ T cells increased with age [30]. In accordance with these previous findings, our study indicates that higher patient age correlates with higher CMV-specific ELISpot responses (but not with higher responses in the QuantiFERON-CMV assay).

In line with our observations in liver transplant recipients, a recent meta-analysis in kidney transplant recipients showed that ELISpot-based monitoring of cellular CMV immunity might be superior to monitoring by the QuantiFERON-CMV assay [11]. The sensitivity and specificity of the CMV-specific ELISpot vs. the QuantiFERON-CMV assay to predict CMV infection was higher overall. These results suggest that CD4+ T cells may be particularly important in controlling CMV-DNAemia. Apart from help for cytotoxic T cells, direct antiviral properties of CMV-specific CD4+ T cells were suggested by Jackson et al. [33] and Swain et al. [34]. Flow cytometric data in a cohort containing 31 solid organ transplant recipients (22 of whom after kidney and one after liver transplantation) indicated that CMV-specific CD4+ T cells were more predictive for protection from CMV-DNAemia requiring treatment than CD8+ T cells [18].

Furthermore, the T-Track CMV measures the response of activated NK and NKT-like cells (contributing to the CMV-specific immune response via bystander activation) [16,26], which could further explain the weaker correlation between the QuantiFERON-CMV assay and T-Track CMV, compared to that of the QuantiFERON-CMV assay and T-SPOT.*CMV*. On the other hand, differences between the two ELISpot assays could be caused by the use of different stimulating antigens. T-Track CMV uses protein antigens, predicted to mimic more closely the response to a natural CMV infection, whereas T-SPOT.*CMV* uses peptide pools [15,26]. Furthermore, results of T-Track CMV were normalized to 200,000 lymphocytes, and those of T-SPOT.*CMV* to 250,000 PBMC, making a direct comparison of SFU results not possible. However, when extrapolating the results of the T-SPOT.*CMV* to SFU per 200,000 lymphocytes, results of the T-SPOT.*CMV* were still higher than the T-Track CMV. Of note, the T-Track CMV tests evaluated either by an algorithm provided by the manufacturer or simply by the calculated median yielded comparable SFU and predictive values. Thus, considering the median value may be sufficient for the evaluation. It should be mentioned that qualitative (i.e., positive/negative) evaluation of ELISpot results—which take into consideration the response to both IE-1 and pp65 antigens—was associated with an increased analytical sensitivity compared to the response of each antigen alone [12,16,35]. However, we here decided to consider IE-1 and pp65 antigens separately in order to directly compare responses to the two CMV antigens from two manufacturers.

Similar to our observation in liver transplant recipients, the above-mentioned study using flow cytometry in solid organ transplant recipients [18] or a study using the QuantiFERON-CMV assay in heart transplant recipients [24]) observed an increase of cellular responses to CMV antigens over time. Apart from CMV infection/reactivation after the end of antiviral prophylaxis, tapering or discontinuation of immunosuppressive drugs—known to suppress antimicrobial immune responses [36]—very likely caused this increase. In the high-risk group (D+/R−) eight out of 14 patients showed CMV replication and thus suffered from primary infection, which should induce T cell immunity. In four out of these eight patients with CMV replication the positivity to the IGRAs even preceded CMV-DNAemia (Table 3). Most likely, viremia below the detection limit of 40 copies/mL was already able to induce cellular immunity. In these four patients the maximum viral load was lower than in patients in whom IGRAs were positive temporally corresponding to onset of CMV-DNAemia. In all three patients with positive CMV-specific ELISpot responses preceding CMV-DNAemia the maximum viral load was below 500 copies/mL; this supports the argument for the protective role of this early CMV-specific cellular immunity.

We used two different cut-off values for the definition of CMV infection/reactivation. When setting the cut-off for CMV-DNAemia at 40 copies/mL, none of the assays was suitable to predict protection from CMV replication. However, when setting a cut-off at 500 copies/mL (substantial CMV-DNAemia) T cell responses were predictive of protection from substantial CMV reactivation. Here, responses of both ELISpot assays appeared superior to that of the QuantiFERON-CMV assay. For example, CMV pp65-specific responses of the T-SPOT.*CMV* at the end of prophylaxis had a specificity of 46% (together with a sensitivity set to 100%) and CMV IE-1-specific responses of the T-Track CMV at month 1 had a specificity of 59% (together with a sensitivity set to 100%). Of note, a previous study by Shin et al. using another ELISpot (CMVspot) [17] set a cut-off at 446 copies/mL (similar to our second cut-off). In line with that previous study, higher responses of the CMV IE-1-specific ELISpot correlated with an absence of CMV-DNAemia in the current study. Apart from the ELISpot assay itself, several important differences between both studies exist: Shin et al. determined CMV-specific immunity prior to liver transplantation, the patients with high or intermediate risk for CMV were treated according to a pre-emptive strategy and thereby experienced earlier CMV infection/reactivation (median of 35 days after transplantation), and positivity to the ELISpot was defined by a stimulation index (and not by SFU). Furthermore, a paper reported the use of the QuantiFERON-CMV assay in liver transplant recipients [37]. This study included 75 patients considered low risk based on pre-transplant CMV serology and all patients received pre-emptive therapy. In that study, a week two result of < 0.1 IU/mL was significantly associated with a higher risk of subsequent CMV-DNAemia (hazard ratio 6.9) [37]. However, due to the other therapeutic regimen and the inclusion of low-risk patients, these data do not compare well to ours.

Finally, in line with our data, it has previously been reported that females display stronger antiviral immune responses, e.g., CMV-specific ELISpot IL-2 and IL-21 secretion was higher in females [32,38].

## 5. Conclusions

This study is the first comparing three commercially available assays to determine CMV-specific cellular immunity in liver transplant recipients. In the current setting, including patients at high and intermediate risk for CMV infection/reactivation, both ELISpot assays appeared similarly predictive of protection from substantial CMV-DNAemia within six months after the end of prophylaxis, whereas the QuantiFERON-CMV was inferior. We defined cut-off values for the various assays and suggest using them for risk stratification. Higher responses could be a marker for protection against CMV, which might only allow close monitoring of the viral load. In the future, the results of randomized intervention studies have to show whether CMV-specific cellular immunoassays are indeed useful for treatment decisions.

## Figures and Tables

**Table 1:** Demographics of 56 liver transplant recipients.

Recipient sex (no., %)	Male	40 (71)
	Female	16 (29)
Age, years (median, range)		55 (20–68)
Disease leading to transplantation (no.)	HCV	11
	HBV	9
	Ethyltoxic Cirrhosis	16
	Autoimmune ^1^	10
	Other	10
Immunosuppressive regimens (no.) ^2^	CNI, MMF	44
	CNI, mTOR	2
	CNI	4
	CNI, MMF, steroids	4
	CNI, mTOR, steroids	1
	CNI, steroids	1
CMV serostatus (no.,%)	D+/R−	14 (25)
	D−/R+	17 (30)
	D+/R+	25 (45)
Patients with CMV-DNAemia post-transplant	D+/R−	8/14 (67)
(no., % of the respective group) ^3^	D−/R+	4/17 (24)
	D+/R+	10/25 (40)
Patients who received antiviral treatment for	D+/R−	6/14 (43)
CMV-DNAemia (no.,% of the respective group) ^3^	D−/R+	3/17 (18)
	D+/R+	9/25 (36)
Allograft rejection (no.)		12

^1^ Primary biliary cirrhosis (*n* = 3) or primary sclerosing cholangitis (*n* = 4) or autoimmune hepatitis (*n* = 3); ^2^ at the end of antiviral prophylaxis; ^3^ CMV-DNAemia ≥40 copies/mL (≥62.4 IU/mL). CMV—*cytomegalovirus*; no.—(absolute) number; D—donor; R—recipient; CNI—calcineurin inhibitor; MMF—mycophenolate mofetil; mTOR—mammalian target of rapamycin.

**Table 2:** Spearman correlation of cytomegalovirus (CMV)-specific cellular assays.

(a) Two Different ELISpot Assays				
Parameter 1	Parameter 2	*r*	*p*	*n*
**IE-1 Lo (Algo)**	**IE OI**	**0.608**	**<0.0001**	**100**
IE-1 Lo (Median)	IE OI	0.606	<0.0001	100
*IE-1 Lo (Algo)*	*IE-1 Lo (Median)*	*0.987*	*<0.0001*	*103*
**pp65 Lo (Algo)**	**pp65 OI**	**0.740**	**<0.0001**	**99**
pp65 Lo (Median)	pp65 OI	0.617	<0.0001	99
*pp65 Lo (Algo)*	*pp65 Lo (Median)*	*0.996*	*<0.0001*	*103*
IE-1 Lo (Algo)	pp65 Lo (Algo)	0.620	<0.0001	103
IE-1 Lo (Median)	pp65 Lo (Median)	0.609	<0.0001	103
IE OI	pp65 OI	0.706	<0.0001	99
(**b**) **ELISpot Assays and QuantiFERON-CMV Assay**				
**Parameter**		***r***	***p***	***n***
IE-1 Lo (Algo)		0.217	0.03	102
IE-1 Lo (Median)		0.217	0.03	102
IE OI		0.387	<0.0001	99
pp65 Lo (Algo)		0.565	<0.0001	102
pp65 Lo (Median)		0.551	<0.0001	102
pp65 OI		0.647	<0.0001	98

The frequency of CMV IE-1- and pp65-specific cells was evaluated in liver transplant recipients by two different IFN-γ ELISpot assays (T-Track CMV and T-SPOT*.CMV*) and the QuantiFERON-CMV assay. Quadruplicate cultures of the T-Track CMV (Lophius Biosciences, Lo, Regensburg, Germany) were evaluated in two ways: either considering an algorithm as specified by the manufacturer (Algo) or using medians. The T-SPOT*.CMV* (Oxford Immunotec, OI, Abingdon, Oxfordshire, UK) uses single cultures. Correlation between results of both manufacturers (Lo (Algo) and OI) using either CMV IE-1 or pp65 as stimulus are marked bold. The correlation between results of the manufacturer Lo using the two ways of evaluation are marked in italics. Panel (**a**) compares various ELISpot assays and includes all values (at the end of prophylaxis and month 1 thereafter). Panel (**b**) compares the ELISpot results with results of the QuantiFERON-CMV assay, also including all values.

**Table 3:** Cellular CMV-specific immune responses and CMV replication in CMV IgG donor (D)+/recipient (R)− liver transplant recipients (*n =* 14).

ID	Age	Sex	IE-1	pp65	IE-1	pp65	IE-1	pp65	IE-1	pp65	QuantiFERON							CMV	Start ^1^	End ^1^	VL
			Lo	Lo	OI	OI	Lo	Lo	OI	OI								Repli-			Max.
			M0	M0	M0	M0	M1	M1	M1	M1	M0	M1	M2	M3	M4	M5	M6	cation			
1	57	F	0	0	1	0	14	19	12	105	0	0.3	0					Y	M1	M2	11,172
2	39	M	0	0	0	0	n.t.	n.t.	n.t.	n.t.	0		0.7					Y	M2	M2	6696
3	54	F	0	0	0	0	0	0	1	0	0	0	100		2			Y	M3	M4	2007
4	55	M	2	0	4	8	0	0	0	0	0	0	17	5	3	7	5	Y	M2	M3	1006
5	47	M	0	0	0	0	1	7	20	40	0	0	7	4			100	Y	M1	M1	381
6	58	F	6	5	3	0	3	57	17	274	0	100	82	78	84	100	100	Y	M3	M5	321
7	66	M	0	1	0	2	1	100	4	76	0	38	4	4	0	8	7	Y	M2	M3	259
8	52	M	0	43	7	51	1	21	13	75	26	39	27	36			27	Y	M1	M1	258
9	43	F	0	0	0	1	0	0	0	0	0	0	0	0	0	0	0	N			
10	51	M	1	1	0	1	0	1	0	0	0	0	0	0	0	0		N			
11	55	M	0	0	0	0	0	0	0	0	0	0	0	0	0	0	0	N			
12	26	M	0	0	0	0	0	0	0	7	0	0	0	0	0		0	N			
13	33	M	1	0	0	0	0	0	0	0	0	0	0				0	N			
14	55	M	0	0	0	1	0	0	0	0	0	0	0	0			0	N			

The data were sorted according to the maximum CMV viral load within six months after the end of prophylaxis (VL Max.), which is given as copies per mL. CMV-specific ELISpot results are presented as spot forming units (IE-1, pp65), either at the end of antiviral prophylaxis (M0) or at month 1 (M1). We performed ELISpot assays from two manufacturers in parallel [T-Track CMV (Lophius Biosciences, Lo) and T-SPOT*.CMV* (Oxford Immunotec, OI)]. Results of the QuantiFERON-CMV assays were indicated as IU/mL. Green labeling means that CMV-specific cellular immunity preceded the detection of CMV DNA. Yellow labeling indicates that CMV-specific cellular immunity and CMV DNA occurred simultaneously. Finally, orange labeling means that CMV-specific cellular immunity occurred after CMV DNA. ^1^ Start and end of CMV replication (given as months after the end of antiviral prophylaxis); n.t.—not tested; M—month; N—no; Y—yes.

**Table 4:** Predictive values of CMV-specific cellular assays in liver transplant recipients.

Test	AUC	Cohort	Time Point	Cut-Off	Sensitivity	Specificity
pp65 OI	0.744	All	M0	142	100%	46%
IE-1 Lo	0.762	All	M1	3.5	100%	59%
QuantiFERON	0.605	All	M1	32	100%	23%
pp65 Lo	0.700	D+/R-	M0	0.5	100%	40%
pp65 Lo	0.722	D+/R-	M1	0.5	100%	44%
pp65 OI	0.722	D+/R-	M1	3.5	100%	44%
QuantiFERON	0.667	D+/R-	M1	19	100%	33%

The value of CMV-specific cellular responses to predict the absence of CMV infection/reactivation was evaluated. For this analysis, the cut-off for CMV infection/reactivation was set at 500 copies of CMV-DNA/mL. It shows either data on all high- and intermediate-risk patients (All) or separately on high-risk patients [donor (D)+/recipient (R)− CMV-IgG serostatus prior to transplantation (D+/R−)]. CMV-specific ELISpot results were considered as spot forming units for CMV IE-1 and pp65 antigens, either at the end of antiviral prophylaxis (M0) or at month 1 (M1). Results of the QuantiFERON-CMV assays were given as IU/mL. The table considers the conditions with the greatest area under curve (AUC) for each assay, as determined by Receiver Operating Characteristic (ROC) curve analyses. A complete data set on the predictive value of all CMV-specific cellular assays is given as Supplementary Tables S2 and S3. OI—Oxford Immunotec (T-SPOT*.CMV*); Lo—Lophius Biosciences (T-Track CMV).

## Data Availability

The data presented in this study are available on request from the corresponding author. The data are not publicly available due to privacy restrictions.

## Supplementary Materials

The following are available online at https://www.mdpi.com/2076-393X/9/2/88/s1, Table S1: CMV-DNAemia in CMV IgG donor (D)+/recipient (R)− liver transplant recipients with viral replication, Table S2: Receiver Operating Characteristic (ROC) curve analyses to define the cellular assay, which is most predictive of protection from CMV infection/reactivation in liver transplant recipients, Table S3: Predictive values of CMV-specific cellular assays in liver transplant recipients.
