# Supplementary material for: Comparison of Three Cellular Assays to Predict the Course of CMV Infection in Liver Transplant Recipients
Source: Vaccines (Basel). 2021 Jan 25;9(2):88. doi: 10.3390/vaccines9020088 (PMC7911226; doi:10.3390/vaccines9020088)
Supplement: Supplementary file 1 [file vaccines-09-00088-s001.pdf]

Supplementary Table 1. CMV-DNAemia in CMV IgG donor +/recipient - liver transplant recipients with viral replication.

ID	M0	M1	M2	M3	M4	M5	M6
**1**	Ø	11172	290	Ø	Ø	Ø	n.t.
**2**	Ø	Ø	6696	Ø	Ø	Ø	n.t.
**3**	Ø	Ø	Ø	1286	2007	Ø	Ø
**4**	Ø	Ø	1006	173	Ø	Ø	Ø
**5**	Ø	381	Ø	Ø	Ø	Ø	Ø
**6**	Ø	Ø	Ø	154	321	50	Ø
**7**	Ø	Ø	259	101	Ø	Ø	Ø
**8**	Ø	Ø	258	Ø	n.t.	n.t.	Ø

This table extends the data on CMV-DNAemia as presented in Table 3 and displays monthly data on DNA load testing. Eight out of 14 CMV IgG donor +/recipient - liver transplant recipients (ID 1-8) showed viral replication after the end of prophylaxis. Viral load is given as copies/ml. M - month after the end of antiviral prophylaxis; Ø - below the detection limit of 40 copies/ml (62.4 IU/ml); n.t. - not tested. Maximum values are highlightened red.

Supplementary Table 2. Receiver Operating Characteristic (ROC) curve analyses to define the cellular assay, which is most predictive of protection from CMV infection/reactivation in liver transplant recipients.

**All**	**Month 0**					**Month 1**				
**Cut-off 40 copies/ml**	**AUC**	**SE**	***p***	**95% CI LL**	**95% CI UL**	**AUC**	**SE**	***p***	**95% CI LL**	**95% CI UL**
IE-1 Lo (Algo)	.517	.084	.836	.353	.682	.575	.090	.427	.399	.751
pp65 Lo (Algo)	.545	.084	.589	.380	.710	.508	.091	.930	.330	.686
IE-1 Lo (Median)	.532	.084	.702	.368	.696	.574	.089	.435	.399	.749
pp65 Lo (Median)	.548	.084	.569	.383	.712	.504	.091	.970	.326	.682
IE-1 OI	.525	.084	.768	.361	.688	.549	.091	.605	.371	.726
pp65 OI	.612	.083	.178	.450	.775	.512	.091	.900	.334	.690
QuantiFERON	.482	.084	.829	.317	.647	.352	.093	.118	.170	.534
**Cut-off 500 copies/ml**	**AUC**	**SE**	***p***	**95% CI LL**	**95% CI UL**	**AUC**	**SE**	***p***	**95% CI LL**	**95% CI UL**
IE-1 Lo (Algo)	.668	.080	.117	.511	.825	.762	.082	.059	.601	.922
pp65 Lo (Algo)	.669	.101	.115	.471	.868	.603	.165	.460	.279	.926
IE-1 Lo (Median)	.698	.081	.065	.539	.856	.772	.082	.050	.610	.933
pp65 Lo (Median)	.675	.103	.103	.473	.877	.610	.163	.427	.291	.930
IE-1 OI	.636	.087	.207	.465	.806	.659	.133	.252	.399	.919
pp65 OI	.744	.074	.023	.599	.888	.687	.136	.177	.420	.955
QuantiFERON	.568	.099	.528	.373	.762	.605	.121	.448	.367	.843

**D+/R-**	**Month 0**					**Month 1**				
**Cut-off 40 copies/ml**	**AUC**	**SE**	***p***	**95% CI LL**	**95% CI UL**	**AUC**	**SE**	***p***	**95% CI LL**	**95% CI UL**
IE-1 Lo (Algo)	.500	.161	1.000	.185	.815	.200	.151	.100	.000	.495
pp65 Lo (Algo)	.375	.152	.439	.077	.673	.233	.160	.144	.000	.547
IE-1 Lo (Median)	.448	.158	.747	.138	.758	.250	.161	.171	.000	.565
pp65 Lo (Median)	.406	.156	.561	.101	.712	.233	.160	.144	.000	.547
IE-1 OI	.250	.133	.121	.000	.511	.100	.114	.028	.000	.323
pp65 OI	.469	.162	.846	.151	.786	.233	.160	.144	.000	.547
QuantiFERON	.438	.158	.699	.128	.747	.200	.151	.100	.000	.495
**Cut-off 500 copies/ml**	**AUC**	**SE**	***p***	**95% CI LL**	**95% CI UL**	**AUC**	**SE**	***p***	**95% CI LL**	**95% CI UL**
IE-1 Lo (Algo)	.513	.179	.944	.162	.863	.667	.187	.480	.300	1.000
pp65 Lo (Algo)	.700	.142	.258	.421	.979	.722	.169	.346	.391	1.000
IE-1 Lo (Median)	.550	.180	.777	.198	.902	.722	.169	.346	.391	1.000
pp65 Lo (Median)	.763	.134	.138	.500	1.000	.722	.169	.346	.391	1.000
IE-1 OI	.375	.172	.480	.037	.713	.500	.205	1.000	.097	.903
pp65 OI	.588	.181	.621	.232	.943	.722	.169	.346	.391	1.000
QuantiFERON	.550	.169	.777	.218	.882	.667	.187	.480	.300	1.000

CMV infection/reactivation was either defined as ≥ 40 copies or ≥ 500 copies of CMV-DNA/ml. The upper panel considers all patients, the lower only high-risk patients [donor (D)+/recipient (R)- CMV-IgG serostatus prior to transplantation (D+/R-)]. CMV-specific ELISpot results were considered as spot forming units for CMV IE-1 and pp65 antigens, either at the end of antiviral prophylaxis (month 0) or at month 1. Patients who developed CMV infection/reactivation between end of prophylaxis and month 1 were excluded from the ROC curve analysis at month 1. Results of the T-Track CMV (Lophius Biosciences, Lo) were calculated using the algorithm provided by the manufacturer (Algo) or using median values. OI means results of the T-SPOT.*CMV* (Oxford Immunotec). Data sets also presented in Table 4 were marked grey. SE - standard error; CI - confidence interval; LL - lower limit; UL - upper limit.

Supplementary Table 3. Predictive values of CMV-specific cellular assays in liver transplant recipients.

Test	AUC	Cohort	Time point	Cut-off	Sensitivity	Specificity	n_c	n_t	n_c/n_t
IE-1 Lo (Algo)	.668	All	M0	5.5	100%	44%	19	51	0.37
pp65 Lo (Algo)	.669	All	M0	284	100%	7%	3	51	0.06
IE-1 Lo (Median)	.698	All	M0	5.5	100%	46%	20	51	0.39
pp65 Lo (Median)	.675	All	M0	342	100%	5%	2	51	0.04
IE-1 OI	.636	All	M0	91	100%	20%	8	50	0.16
pp65 OI	.744	All	M0	142	100%	46%	19	50	0.38
QuantiFERON	.568	All	M0	9.9	100%	22%	10	56	0.18
IE-1 Lo (Algo)	.762	All	M1	3.5	100%	59%	25	48	0.52
pp65 Lo (Algo)	.603	All	M1	470	100%	3%	1	48	0.02
IE-1 Lo (Median)	.772	All	M1	4.5	100%	59%	25	48	0.52
pp65 Lo (Median)	.610	All	M1	471	100%	3%	1	48	0.02
IE-1 OI	.659	All	M1	153	100%	15%	6	46	0.13
pp65 OI	.687	All	M1	311	100%	20%	8	45	0.18
QuantiFERON	.605	All	M1	32	100%	23%	9	47	0.19
IE-1 Lo (Algo)	.513	D+R-	M0	4.0	100%	10%	1	14	0.07
pp65 Lo (Algo)	.700	D+R-	M0	0.5	100%	40%	4	14	0.29
IE-1 Lo (Median)	.550	D+R-	M0	5.0	100%	10%	1	14	0.07
pp65 Lo (Median)	.763	D+R-	M0	1.5	100%	30%	3	14	0.21
IE-1 OI	.375	D+R-	M0	5.5	100%	10%	1	14	0.07
pp65 OI	.588	D+R-	M0	30	100%	10%	1	14	0.07
QuantiFERON	.550	D+R-	M0	13	100%	10%	1	14	0.07
IE-1 Lo (Algo)	.667	D+R-	M1	0.5	100%	33%	3	12	0.25
pp65 Lo (Algo)	.722	D+R-	M1	0.5	100%	44%	4	12	0.33
IE-1 Lo (Median)	.722	D+R-	M1	0.5	100%	44%	4	12	0.33
pp65 Lo (Median)	.722	D+R-	M1	0.5	100%	44%	4	12	0.33
IE-1 OI	.500	D+R-	M1	2.5	100%	33%	3	12	0.25
pp65 OI	.722	D+R-	M1	3.5	100%	44%	4	12	0.33
QuantiFERON	.667	D+R-	M1	19	100%	33%	3	12	0.25

It was analyzed how different CMV-specific cellular assays predicted the absence of CMV infection/reactivation. For this analysis, the cut-off for CMV infection/reactivation was set at 500 copies of CMV-DNA/ml. The table considers all cellular in vitro assays. It shows either data on all high and intermediate risk patients (All) or separately on high risk patients [donor (D)+ recipient (R)- CMV-IgG serostatus prior to transplantation]. CMV-specific ELISpot results were considered as spot forming units for CMV IE-1 and pp65 antigens, either at the end of antiviral prophylaxis (M0) or at month 1 (M1). Results of the T-Track CMV (Lophius Biosciences, Lo) were calculated using the algorithm provided by the manufacturer (Algo) or using median values. OI means results of the T-SPOT.*CMV* (Oxford Immunotec). Results of the QuantiFERON-CMV assay were given as IU/ml. Data sets also presented in Table 4 were marked grey. AUC - area under curve; n_c - number of patients exceeding the cut-off value; n_t - total number of patients.
